# Collaborative risk assessment and management planning in secure mental health services in England: protocol for a realist review

**DOI:** 10.1136/bmjopen-2025-099747

**Published:** 2025-05-27

**Authors:** Catherine Jeynes, Naomi Clifford, Ian Callaghan, Naomi Thorpe, Brian Crosbie, Katrina Forsyth, Seena Fazel, Daniel Whiting

**Affiliations:** 1 Nottinghamshire Healthcare NHS Foundation Trust, Nottingham, UK; 2 Rethink Mental Illness, London, UK; 3 University of Nottingham, Nottingham, UK; 4 The University of Manchester, Manchester, UK; 5 Department of Psychiatry, University of Oxford, Oxford, UK; 6 Oxford Health NHS Foundation Trust, Oxford, UK

**Keywords:** Forensic psychiatry, Review, Vulnerable Populations, Risk management

## Abstract

**Abstract:**

**Introduction:**

Secure mental health pathways are complex. They are typically based around secure hospitals, but also interface with justice agencies and other clinical services, including in the community. Consideration of risk is fundamental to clinical care and to decisions relating to a patient’s stepwise journey through the pathway. Patient autonomy and involvement in decision-making are policy priorities for health services. However, improving collaboration in risk-related decisions in secure services is complicated by potential issues with insight and capacity and the necessary involvement of other agencies. In addition, although some collaborative approaches are feasible and effective, their impact, mechanisms and the contexts in which they work are not well understood. Therefore, using realist methodology, this review will outline what works, for whom, why and under what circumstances in terms of collaborative risk assessment and management in secure services.

**Methods and analysis:**

The review will consist of four stages: (1) Development of an initial programme theory to explain how and why collaborative risk assessment and management works for different groups of people, (2) search for evidence, (3) data selection and extraction and (4) evidence synthesis and development of a final programme theory. Our initial programme theory will be informed by an informal search of the literature and consultation with experts and patient and public involvement and engagement representatives. Following this, our formal literature search will include both the published and unpublished literature. During full text screening, each document will be assessed according to the principles of rigour and relevance and, if included, data will be extracted and synthesised to refine the programme theory.

**Ethics and dissemination:**

This protocol is for a review of published literature and so does not require ethical approval. The main output will be the final programme theory. Remaining gaps will inform planned future work to further refine the theory using mixed methods. Our dissemination strategy will be codeveloped with our public and patient involvement group and will include publishing findings in a peer-reviewed journal and presenting findings at relevant professional conferences, as well as engaging patient, carer and clinician groups directly.

**STRENGTHS AND LIMITATIONS OF THIS STUDY**

- The chosen realist methodology is well suited to assessing the effectiveness of interventions in complex healthcare settings, where there is likely to be variation in implementation and effectiveness.
- A strong public and patient involvement component is embedded throughout the research cycle, from conceptualisation to dissemination, ensuring the work and delivery of its outputs is anchored in the expertise of patients and carers with lived experience of these settings.
- The subject expert group advising the review is made up of a wide range of experts in the field, who are well placed to be able to assess the validity of the emerging programme theory as it relates to clinical practice.
- The wider generalisability of the review’s findings and recommendations may be limited by the focus on services in England, which is the focus of this review.
- The realist review relies on existing literature, which may not always provide sufficient details on crucial elements of a programme theory, such as mechanisms and contextual factors.

## Introduction and background

 Secure mental health services are complex and resource-intensive, often providing long-term care for people with multiple severe mental health and comorbid physical health needs. The secure mental health pathway in England comprises the secure hospital system, which, in England, exists across three security levels (low, medium and high) and consumes around a fifth of the annual mental health budget,^1^ and community forensic mental health teams. The pathway includes independent sector secure hospitals which may be commissioned to provide care as part of National Health Service (NHS)-led Provider Collaboratives.^2^ Secure services in England also interface with many other services at different steps in the pathway, especially general mental health services, mental health services in prison and aligned justice agencies.

Provision of care within the secure pathway in England is guided largely by risk. Throughout, we refer to risk as per the Department of Health’s definition, the ‘likelihood, imminence and severity of a negative event occurring’.^3^ Most commonly in secure services, the risk considered is of harm to others.^4^ However, many other risks are also considered, such as self-harm, suicide, disengagement, mental health relapse or other vulnerability. These risks and linked restrictive decisions are a uniquely prominent aspect of care in these settings and encompass the complex dual elements of individual need and public safety. Consideration of risk is integral to admission, discharge and stepwise progress through the secure pathway. This can include the most acute, restrictive forms of management, for example, seclusion or long-term segregation, through to decisions around community leave, hospital security, discharge and community supervision.^5^

Considering the pivotal role that risk assessment and management play in progression through the secure mental health pathway, it is important that patients are offered the opportunity to engage in meaningful collaboration with professionals in creating those risk assessments and management plans. This importance is highlighted by the goal of the NHS Long Term Plan to maximise patient-centred care, autonomy and collaboration in decision-making^6^ and in the context of the UK’s Independent Review of the Mental Health Act^7^ which set out a number of recommendations for frameworks to become more responsive to patients’ needs and wishes, protect patients’ rights and improve patients’ ability to contribute towards choices even in restrictive circumstances.

There is a need for special consideration of how such collaboration in risk assessment and management planning can be achieved in the complex pathway of secure mental health services. Optimising patient-centred care in secure settings, by making assessment of risk and aligned management decisions a collaborative process, has specific challenges given the potential issues with insight and capacity.^8^ This is complicated further by various other individual and environmental contextual factors that may impact collaboration, as well as the involvement of a combination of agencies which may have competing needs and priorities.

Despite this complexity, a collaborative approach has been recommended for several years.^3^ The extent to which it has been implemented in practice, however, is not known. For example, there remains limited understanding of the adoption and impact of the 2014–2015 and 2015–2016 Commissioning for Quality and Innovation frameworks, which sought to increase patient collaboration in risk assessment by delivering a joint education package to staff and patients.^8^

In general mental health populations, evidence syntheses suggest that shared decision-making may be more suitable when applied to long-term decisions,^9^ which characterise those in the secure pathway, but generally there is low certainty about the effects and a need for further research.^10^ In secure settings, such collaboration can potentially improve broad outcomes, from person-level improvements in self-agency, quality of life and ability to implement behavioural change^11^ to longer-term outcomes such as length of stay, reoffending and readmission.^12^ Some primary studies indicate the feasibility of engaging patients in identifying risks and needs and that incorporating patient self-assessment of risk may improve the prediction of re-offending.^13^,^15^

There have been two systematic reviews of patient collaboration in risk assessment in secure settings, which have identified promise in terms of the feasibility of implementing collaborative risk assessment and some positive outcomes arising from the inclusion of patients in risk assessment and management.^12 16^ However, these reviews focused narrowly on violence risk assessment and quantitative studies and emphasised patient collaboration only (rather than also considering families and carers). The specific importance of carer collaboration throughout secure mental health service involvement has previously been highlighted,^17^ and a review of evidence pertaining to carer collaboration in risk assessment remains a key evidence gap. Finally, systematic reviews excel in synthesising evidence for or against an intervention and, while this is valuable, they are unable to delve more deeply into why or how the intervention might work or how it might work differently for different groups. Given the inherent complexity of patient collaboration in risk assessment and management, a realist review is well suited to this question.^18^ Rather than assessing the overall efficacy of an intervention, realist reviews focus instead on understanding the underlying mechanisms and contexts through which an intervention might work and on differentiating how these mechanisms and contexts might operate within different subgroups of patients.^19^ This approach, in conjunction with wide stakeholder involvement, can therefore achieve outputs that are both theoretically anchored and practically relevant.

The realist methodology also allows for a broader scope of evidence to be included in the review. The two existing systematic reviews focused on collaboration in violence risk assessment reported a low number of studies eligible for inclusion (five in one review and three in the other). Whereas a systematic review includes only randomised controlled trials (RCTs), quasi-RCTs and controlled trials, a realist review necessarily draws on a wider pool of evidence to understand why and how an intervention works.^19^ This may mitigate the risk of low numbers of included evidence pieces. However, should the review find that there are still low numbers of relevant texts for inclusion, it will be able to comprehensively outline the remaining gaps in the evidence and in our current understanding of how collaboration in risk assessment and management works in secure services.

The aims of this review are therefore to use realist methodology to understand: (1) what approaches to collaborative risk assessment and management in secure mental health settings have been described and (2) how, in what circumstances and why they work for patients, carers and healthcare professionals. This review forms a part of a larger programme of research that will provide recommendations and best practice guidance to inform the way risk is collaboratively assessed and managed in secure services in England.

## Methods and analysis

### Stakeholder involvement

#### Patient and public involvement

Patient and public involvement and engagement (PPIE) is embedded throughout by the formation of a PPIE group consisting of 12 people with lived experience, either current or past, of secure mental health services as patients and carers.

Participants were identified through existing Rethink Mental Illness channels, national lived experience and clinical networks and from other projects in secure services. All participants received an information sheet, gave informed consent and participated in informal calls to assess well-being and readiness prior to joining the group. The PPIE group includes people who have entered the secure pathway via different routes, including transfer from prison, and there is a good geographical spread across England.

The PPIE group will meet online approximately three times for 2 hours throughout the review process. There will be two facilitators and one note-taker from Rethink Mental Illness. Meetings will begin with a group agreement to remind people of safety, for the comfort of participants and for safeguarding purposes. Optional debrief meetings will be held 2–3 days after group meetings to allow participants to make any further contributions and to check on well-being.

Agreements have been made with the clinical teams of participants in hospital that ward staff can support the patient’s participation in PPIE meetings by arranging for secure ward laptops to be used in private areas of the ward. Communication from the coordinating team at Rethink Mental Illness therefore goes to both the group member, if they have an email address, and to the contact on the ward, who is asked to print out any prereading materials, such as agendas, previous minutes or background reading for the meeting.

The PPIE group will support the research team in refining the evidence search by giving feedback on the list of search terms, reviewing and developing ‘if-then’ statements and contributing to and sense-checking context-intervention-mechanism-outcome (CIMO) configurations (CIMOCs). They will offer perspectives on the emerging themes and where to target additional searches, and support the team in developing and refining the programme theory (PT), which aims to explain how and why collaborative risk assessment and management works for different groups of people. They will also be able to contribute to the research after meetings by providing feedback via email. Finally, once the PT is finalised and the review process is complete, the PPIE group will also input into the coproduction of outputs and their dissemination.

#### Subject expert group

The subject expert group (SEG) comprises 24 stakeholder representatives. It includes representation of all levels of hospital security, community forensic services, women’s services, specialist settings including for intellectual disability and autism, NHS and independent sector providers, the Royal College of Psychiatrists, the British Psychological Society, the Ministry of Justice and the First-tier Tribunal. The group includes a range of multiprofessional frontline clinicians, in addition to our PPIE lead and an additional carer representative. The group was initially formed via the professional network of the research team and was subsequently iteratively populated according to gaps identified by the group itself in initial meetings.

The group will meet together with the research team approximately three times throughout the realist review process, with the possibility of ad hoc meetings/workshops taking place as required. The SEG will provide input during key stages of the review process including but not limited to: (1) the initial formation of the PT, to assess its validity when compared with current clinical practice, (2) the development of literature search terms and suggestions for additional searches as needed, (3) the processes of data synthesis including the development of ‘if-then’ statements and development of CIMOCs, (4) development of the final PT and fine-tuning the theory to accurately reflect current practice and (5) plans for dissemination of the results of the review.

### Realist methodology

When conducting this review, we will follow the RAMESES guidelines and standards for realist reviews^20^ and these results will populate a PT. This PT will then be used to inform subsequent mixed methods work (outside of the scope of this protocol) focused on examining whether and how these collaborative approaches are implemented across England, to understand how this can improve. The review has been preregistered on PROSPERO (CRD42024607194) with a start date of October 2024, ending in September 2025. The feasibility of the search and data extraction/synthesis process is supported by the inclusion of dedicated funded time included as part of the research grant, oversight by the project leadership team and assistance of an information specialist.

### Equality impact assessment

Prior to starting the review, an equality impact assessment (EqIA) will be undertaken to ensure that the review is inclusive and does not inadvertently disadvantage minoritised groups. When carrying out the EqIA, we will consider each of the minoritised groups in turn (age, disability, sex, pregnancy and maternity, gender reassignment, sexual orientation, race/ethnicity, marriage and civil partnership and religion and belief) and will consider how the review might impact each group considering the available literature and evidence around each protected group in relation to the research area.^21^ The subsequent processes will be guided by the findings of that assessment.

### Study design

**Figure 1:**
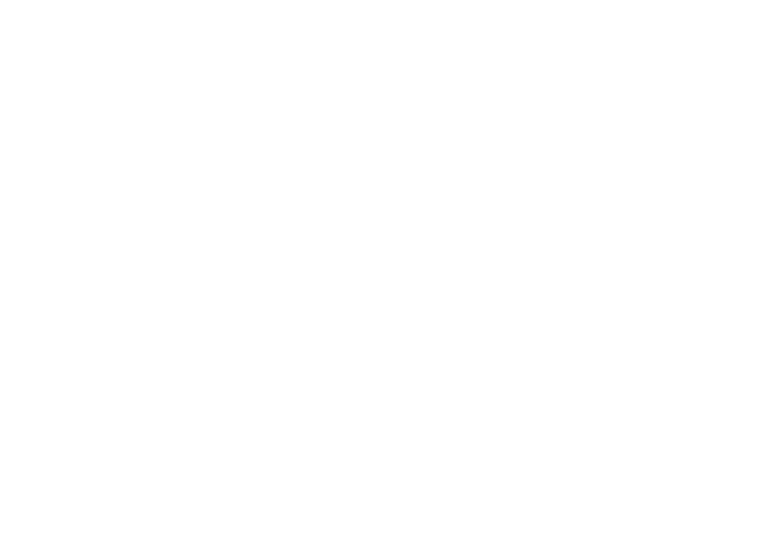
Overview of the stages of the realist literature review. Adapted from Power et al^23^

The realist review will consist of several stages, which are repeated iteratively as necessary to populate the developing PT. Figure 1 outlines the realist review process.^22 23^

#### Stage 1: developing an initial PT

The first step of the review is to develop an initial PT. This will be created through the professional knowledge and expertise of our research team and through initial perusal of documents held in the research team’s personal collections. Once the first draft of the PT has been created, this will be reviewed and refined by the SEG and the PPIE group.

Using the standard realist formulation of context+mechanism = outcome,^22^ with the addition of intervention resource (C+I+M=O), initial discussions among the research team will focus on identifying the intervention components used to facilitate collaboration in risk assessment and management and the contexts in which these interventions occur, alongside any outcomes observed in practice. The initial perusal of documents will identify these components of the CIMO configurations (CIMOCs) but will also explore potential generative mechanisms around why the outcomes occurred in each context. These initial theories will take the form of ‘if-then’ statements; ‘if context X is present, then when intervention A is carried out, outcome Y will occur because of mechanism B’. The creation of ‘if-then’ statements is an initial step towards understanding, organising and classifying the available data and can then lead on to the creation of CIMOCs.^24^

#### Stage 2: search for evidence

After the initial PT is created, we will then proceed to a literature search. Our information specialist will conduct the searches, which will include both the published and grey literature. We will use a comprehensive list of search terms, extracted from the initial PT, to ensure that we include all relevant documents. When we have developed a list of search terms, we will send this to both the SEG and the PPIE group for review.

The published literature search will focus primarily on the following databases: Ovid Medline ALL, Ovid EMBASE, ProQuest APA PsycInfo, and for the retrieval of grey literature, the following sources will be searched: BASE, CORE, King’s Fund, Nuffield Trust, TRIP Database, EthOS, OATD and Google Scholar. To ensure that the search is as comprehensive as possible, we will also use purposive search strategies such as snowballing (citation searches).^25^

#### Stage 3: data selection and extraction

To determine whether a document should be included in the review, two members of the research team will screen the titles and abstracts of all documents retrieved by the searches. The title and abstract screening process will be supported through use of RAYYAN software. Documents will be selected for full text screening if they meet the inclusion criteria. These are captured in table 1 and are intentionally broad to provide as much relevant data as possible.

**Table 1:** Inclusion criteria

Population	Patients, carers and professionals within the secure mental health pathway, including people transferred from prison to hospital, community forensic services, all secure hospital settings and professionals from any relevant aligned agencies (such as the Ministry of Justice).
Intervention	Collaborative risk assessment/management planning defined as any approach that seeks to actively involve patients and/or their carers to the extent that they wish to be involved in assessments of risk and/or the decisions and management plans that are directly linked to that assessment of risks. Risk is defined as per the Department of Health, as the ‘likelihood, imminence and severity of a negative event occurring’. There is no limit placed on that negative event for this review, although most typically in this context this will be of harm to self or others.
Document type/study design	No restriction
Other	This review primarily focuses on collaborative risk assessment and management strategies used in England; however, where the literature pertaining to other regions is judged by the research team to provide evidence relevant to populating aspects of the PT, this can be included.

PT, programme theory.

The lead reviewer will then screen the full text of those documents that have passed the title/abstract screen. At full text screening, 20% of the documents will be screened by a third reviewer to provide an inter-rater comparison. Any discrepancies will be discussed and resolved by the wider research team.

During the full text screening, the lead reviewer will assess each document according to the principles of relevance and rigour.^22^ Relevance in realist methodologies is defined as ‘whether (the data) can contribute to theory building and/or testing’.^20^ In making decisions about whether a particular document is relevant, the reviewer will ask two questions.^26^ First, they will ask whether the document is relevant to the topic of collaborative risk assessment and management in secure mental health services. Second, they will ask whether the document provides evidence that is relevant to the development of the PT. The first question will be determined during full text screening, whereas the second will be reassessed as the PT develops. This reassessment is necessary as previously excluded evidence may become relevant during theory development. Any papers that might possibly be relevant in future will be assigned to the ‘reserve list’ to allow for this reassessment if needed. The RAMESES quality standards for realist reviews define rigour as ‘whether the method used to generate that particular piece of data is credible and trustworthy’.^20^ The RAMESES guidelines do not recommend any particular checklist; however, when considering text or data for inclusion, the following questions will be asked: are the data likely to be biased? Are the data/theory described in the text critically analysed? Do the data/theory derive from real-world examples or are they based on theoretical speculation? Are the data gathered in depth over time or do they represent a brief glimpse of the theory/situation being assessed? Is it safe to generalise from this data? Are the data/theory being described also seen in other sources (triangulation)? Are the data/theory consistent with the experience of the research team and the stakeholder groups (SEG and PPIE group)?^26 27^

When considering whether to include documents on the basis of rigour and relevance, relevance will be valued over rigour. However, we will ensure that subsequent work endeavours to substantiate the contribution of any data that are not sufficiently rigorous and, when reporting the results of the realist review, we will ensure that we highlight any areas that are supported by data of questionable rigour.

Data extraction will focus on drawing out information relevant to the initial PT. For each outcome (O) identified in the PT components, we will gather information on the intervention activities (I), the mechanism (M) by which this outcome occurred and the context (C) under which that mechanism will lead to the desired outcome.^22^ Data extraction templates will be developed in Microsoft Excel that capture this CIMO data. These templates will then be piloted and refined following discussion with the research team to ensure that the forms capture all relevant information. The information captured will take the form of sections of text extracted from relevant documents. Data extraction templates will be used in conjunction with NVivo (V.12) to support the organisation, coding and retrieval of data from the primary sources.

#### Stage 4: evidence synthesis and development of final PT

We will analyse the data using realist logic; creating CIMOCs that will then be used to refine our initial PT.

Sections of relevant text will be highlighted and coded in NVivo. For each of these sections, we will ask a series of questions in order to determine whether it is best described as a context, intervention, mechanism or outcome, whether it fits within a pre-existing CIMOC or whether it is the first element of a new CIMOC. We anticipate that this will involve comparison across sources to fully elucidate the context, intervention, mechanism or outcome and how it fits within the CIMOC. For example, information regarding mechanisms from one source might well be useful in understanding the link between context and outcome in another. As the CIMOCs develop, we will add them to the initial PT, asking how this CIMOC relates to both the other CIMOCs and to the overall PT in terms of where it fits within the theory and whether the theory itself needs to be revised. Where there are any gaps in the evidence from secure mental health settings, we will use the ‘reserve list’, which will hold papers that provide relevant insights into collaborative risk assessment in general adult psychiatric services. When reporting the PT, any areas which rely on papers from the reserve list will be highlighted.

As the PT develops, we will consult the SEG and the PPIE group at regular intervals for the purposes of sense-checking the theory as it compares to their experience. As an example of this, for the SEG and PPIE group meetings, the research team will devise clear CIMO statements, comprising the ‘if’-‘then’ logic. Effort will be made to make these statements understandable in the context of people's experience. This will enable members to deliberate and contribute further suggestions for the refinement of the CIMOs and PT.

If needed, we will conduct further searches to fully understand and develop the relationships between the contexts, mechanisms and outcomes identified. Throughout the analysis, we will move iteratively between identifying subsections of data as context, intervention, mechanism or outcome, developing the CIMOCs, refining the overall PT and conducting further searches.

In the last stage of the review process, we will ensure that we consult the SEG and the PPIE group when we have the final draft of the PT. They will have been consulted at each stage of the PT development; when forming the initial PT, when defining the review search terms and when developing CIMOCs using 'if-then' statements. However, this final consultation will allow the SEG and PPIE group to fine-tune the PT to ensure that it aligns with the experiences of both those working in practice and those receiving care.

## Ethics and dissemination

This protocol is for a review of the published literature and so does not require ethical approval.

The primary output will be a PT outlining the various available methods of collaborative risk assessment and management, the outcomes that result from these approaches, the context in which those outcomes have been observed and the mechanisms through which the outcomes are achieved. The development of the PT will also highlight any gaps in the literature, which can be addressed in subsequent mixed methods work to be undertaken by the research team. We will also aim to publish the review in a peer-reviewed journal article and present findings at relevant professional conferences.

The next stages of the work will use surveys and interviews to further develop the findings of the review. This mixed methods work package will be developed in conjunction with the PPIE group and the SEG and will assess the current usage of collaborative risk assessment and management strategies in England and the observed contexts, mechanisms and outcomes associated with these practices. We anticipate the results of the mixed methods work packages assessing the current extent of collaboration in risk assessment and management in secure services to be of more interest to patients and the general public. Accordingly, we will focus our patient-facing and carer-facing dissemination strategies on this stage of the project. However, we expect that these dissemination strategies will include summaries of the realist review and so are summarised here. Patient-facing and carer-facing summaries will be co-produced with PPIE groups with the intention that these can be made directly available in relevant clinical settings. Posters and leaflets will be prepared for patient noticeboards using a ‘You said, we did’ framework. Finally, wider communication for patient/public audiences such as with online blogs will be planned with the PPIE groups.
