# Supplementary material for: Collaborative risk assessment and management planning in secure mental health services in England: protocol for a realist review
Source: BMJ Open. 2025 May 27;15(5):e099747. doi: 10.1136/bmjopen-2025-099747 (PMC12121565; doi:10.1136/bmjopen-2025-099747)
Supplement: Supplementary file 1 [file bmjopen-2025-099747.reviewer_comments.pdf]

PEER REVIEW HISTORY

BMJ Open publishes all reviews undertaken for accepted manuscripts. Reviewers are asked to complete a checklist review form and are provided with free text boxes to elaborate on their assessment. These free text comments are reproduced below.

ARTICLE DETAILS

Title (Provisional)

Collaborative risk assessment and management planning in secure mental health services in England: protocol for a realist review

Authors

Jeynes, Catherine; Clifford, Naomi; Callaghan, Ian; Thorpe, Naomi; Crosbie, Brian; Forsyth, Katrina; Fazel, Seena; Whiting, Daniel

---

VERSION 1 - REVIEW

**Reviewer**	**1**
**Name**	**Clibbens, Nicola**
**Affiliation**	**Northumbria University**
**Date**	**24-Feb-2025**
**COI**	**None**

Thank you for presenting a very well written realist evidence synthesis protocol paper. I have very minor suggestions for edits before publication.

1. on page 10 line 16; please clarify what is meant by "each subsection of data". It would be helpful to distinguish the synthesis process more clearly from the data extraction process presented in the previous section.

2. The stakeholder engagement is mostly clearly outlined and fits well with realist evidence synthesis methods. The process of validating the programme theory could be more explicitly linked to engagement with stakeholders. Please see page 10 line 30 and page 11 lines 9-16 where this could be added.

3. I was surprised that you had not cited/applied recent realist approach (Dada et al 2023) to relevance and rigour assessment. https://doi.org/10.1002/jrsm.1630.

**Reviewer**	**2**
**Name**	**Weich, Scott**
**Affiliation**	**University of Sheffield, School of Health and Related Research**

**Date**	**28-Mar-2025**
**COI**	**None**

Thank you for asking me to look at this review protocol.

This is an important study and the protocol will be of interest. However, I think there are elements that could be clarified and strengthened.

In particular, it took me a little while to appreciate that the focus of the study (and the realist question) is patient involvement in risk assessment and management. There's a bit of slippage in terminology and words like 'collaboration' and 'involvement' are used interchangeably. This is an very important topic and I think the early parts of the manuscript might be more impactful if the focus and aims were made a little clearer. Likewise, the PPIE aspects of the research could be a little clearer (and more consistent) especially as this feels like a real strength of the research.

In terms of describing the study methods, I note that the review appears to conclude with evidence synthesis rather than further iteration of the PTs together with stakeholders and lived experience experts. If that's the case, then this is fine. But I wondered if this is planed but not fully described here?

One further reflection is that it's not altogether clear what's meant by 'secure' services, and which of the NHS and privately provided services might be in scope? Does this include only medium security services, or does it extend to high and/or low security forensic services?

Finally, I wonder if the limitations of the research might extend beyond a single bullet point. Realist reviews have much to recommend them but they are not without their critics.

**Reviewer**	**3**
**Name**	**Van Berkel, Jantien**
**Affiliation**	**Utrecht University, Interdisciplinairy Social Science: Public Health**
**Date**	**30-Mar-2025**
**COI**	**None**

I appreciate the opportunity to review this study protocol and would like to commend the authors for developing an interesting and well-structured research plan. The realist approach chosen for this study is particularly promising, as it has the potential to generate relevant and nuanced findings regarding secure mental health services. I have only minor remarks, which I hope will be helpful in further refining and preparing the study.

Introduction

Terminology Consistency:

The introduction would benefit from greater consistency in terminology, particularly concerning the description of secure mental health services. The title refers to "secure mental health services," whereas the introduction mentions "secure psychiatric services." Later, it becomes clear that secure services encompass different types of facilities. Ensuring clarity and consistency in these terms will help the reader better understand the scope of the study.

Strengthening the Justification for a Realist Review:

The last three paragraphs of the introduction argue why a realist review is necessary. While I agree that a realist approach is well-suited to this topic and will yield valuable insights, the reasoning as presented could be made more robust. The authors mention that systematic reviews exist but that evidence remains scarce. However, scarce evidence alone does not necessarily justify conducting another review, as a review does not expand the empirical evidence base per se. Instead, a realist review provides a different analytical lens to examine existing literature, helping to unravel underlying mechanisms, relevant subgroups, and contextual factors. If the literature does not provide these insights, the review will help identify knowledge gaps in these areas. The justification should focus on the need for a nuanced understanding of how interventions work, under what circumstances, and for whom. Additionally, it is worth highlighting that the interventions studied are complex, as are the systems in which they are embedded. The authors might consider referencing Malcolm Williams' work on complex systems and realism to strengthen this argument.

Methods and Analysis

Subheading "Patient and Public Involvement":

I found this heading somewhat surprising, as the study does not only involve patients and the public but also experts, including policymakers and researchers. A more inclusive term, such as "Stakeholder Involvement" or "Stakeholder Consultation," may better reflect the breadth of participation. Furthermore, if not already included, I recommend involving frontline mental health professionals, as their unique perspective could significantly enrich the study.

Ethical Considerations:

I did not find explicit ethical considerations regarding the involvement of patients, the public, and experts. Given the participatory nature of the study, it would be beneficial to outline the ethical framework governing these interactions, including issues such as informed consent and data protection.

Details on Study Phases:

Under "Study Design," in the Methods and Analysis section, more concrete details are needed for Phases 2, 3, and 4. It is currently unclear what specific steps will be taken, and how relevance and rigour will be assessed for example. Additionally, given the likely high number of articles retrieved, the title and abstract screening process will be time-

consuming. The authors should consider discussing potential strategies for managing this workload, such as using screening tools like Rayyan.

Strengths and Limitations

To further reflect on the study protocol, I suggest discussing the strengths and limitations (currently only outlined in the abstract) in some more detail.

Strengths: One of the major strengths of this study is its use of a realist approach, which allows for a deeper exploration of the mechanisms driving outcomes in secure mental health services. This approach is particularly useful given the complexity of the interventions and the systems in which they operate. Additionally, the inclusion of multiple stakeholder perspectives—including patients, professionals, and policymakers—enhances the study's potential to generate findings that are both theoretically and practically relevant.

Limitations: One possible limitation is the reliance on existing literature, which may not always provide sufficient details on mechanisms and contextual factors. While the authors acknowledge this by incorporating stakeholder consultation, further discussion on how potential gaps in the literature will be addressed could be beneficial. Additionally, given the large number of studies likely to be screened, the feasibility of the review in terms of time and resources should be considered, along with potential mitigation strategies.

I have provided further specific comments within the uploaded PDF. I appreciate the efforts of the authors in developing this study protocol and look forward to seeing its results.

**** **The reviewer provided a marked copy with additional comments. Please contact the publisher for full details.** ****

VERSION 1 - AUTHOR RESPONSE

Reviewer: 1

Dr. Nicola Clibbens, Northumbria University

Comments to the Author:

Thank you for presenting a very well written realist evidence synthesis protocol paper. I have very minor suggestions for edits before publication.

1. on page 10 line 16; please clarify what is meant by "each subsection of data". It would be helpful to distinguish the synthesis process more clearly from the data extraction process presented in the previous section.

Our response:

Thank you, we have clarified this statement as below.

Study Design, Stage 4, page 13, paragraph 2:

Sections of relevant text will be highlighted and coded in NVivo. For each of these sections~~subsection of data~~ we will ask a series of questions in order to determine whether it is best described as a context, intervention, mechanism, or outcome and which CIMOC it fits best with.

2. The stakeholder engagement is mostly clearly outlined and fits well with realist evidence synthesis methods. The process of validating the programme theory could be more explicitly linked to engagement with stakeholders. Please see page 10 line 30 and page 11 lines 9-16 where this could be added.

Our response:

We have now detailed how we will interface with stakeholders to develop the programme theory and agree that this is a helpful clarification.

Study Design, Stage 4, page 13, paragraph 3:

As the programme theory develops, we will consult the Subject Expert Group (SEG) and the PPIE group at regular intervals for the purposes of sense-checking the theory as it compares to their experience. As an example of this, ~~F~~for the Subject Expert Group (SEG) and PPIE group meetings, the research team will devise clear CIMO statements, comprising of 'if' – 'then' logic. Effort will be made to make these statements understandable in the context of peoples' experience. This will enable members to deliberate and contribute further suggestions for refinement of the CIMOs and PT.

3. I was surprised that you had not cited/applied recent realist approach (Dada et al 2023) to relevance and rigour assessment. https://doi.org/10.1002/jrsm.1630.

Our response:

We thank the reviewer for highlighting this reference, which we now cite in our expanded description of assessing rigour and relevance.

Stage 3: Data selection and extraction, page 10, paragraph 3:

During the full text screening, the lead reviewer will assess each document according to the principles of relevance and rigour~~rigour and relevance~~.[23] Relevance in realist methodologies is defined as "whether [the data] can contribute to theory building and/or testing"[21]. In making decisions about whether a particular document is relevant, the reviewer will ask two questions.[27]Firstly they will ask whether the document is relevant to the topic of collaborative risk assessment and management in secure mental health services. Secondly they will ask whether the document provides evidence that is relevant to the development of the programme theory. The first will be determined during full text screening, whereas the second question will be

Formatted: Font: (Default) Aptos, 11 pt, Italic, Complex Script Font: Arial, 11 pt, Italic, English (United Kingdom)

Formatted: Font: (Default) Aptos, 11 pt, Italic, Complex Script Font: Arial, 11 pt, Italic, English (United Kingdom)

Formatted: Font: (Default) Aptos, 11 pt, Italic, Complex Script Font: Arial, 11 pt, Italic, English (United Kingdom)

Formatted: Font: (Default) Aptos, 11 pt, Italic, Complex Script Font: Arial, 11 pt, Italic, English (United Kingdom)

Formatted: Font: (Default) Aptos, 11 pt, Italic, Complex Script Font: Arial, 11 pt, Italic, English (United Kingdom)

Formatted: Font: (Default) Aptos, 11 pt, Italic, Complex Script Font: Arial, 11 pt, Italic, English (United Kingdom)

Formatted: Font: (Default) Aptos, 11 pt, Italic, Complex Script Font: Arial, 11 pt, Italic, English (United Kingdom)

reassessed as the programme theory develops. This reassessment is necessary as previously excluded evidence may become relevant during theory development. Any papers that might possibly be relevant in future will be assigned to the "reserve list" to allow for this reassessment if needed. This will include any papers that are relevant to collaborative risk assessment but are conducted within adult psychiatric services rather than forensic mental health services. The RAMESES quality standards for realist reviews define rigour as "'whether the method used to generate that particular piece of data is credible and trustworthy".[21] The RAMESES guidelines do not recommend any particular checklist, however, when considering text or data for inclusion the following questions will be asked: Is the data likely to be biased? Is the data/theory described in the text critically analysed? Does the data/theory derive from real-world examples or is it based on theoretical speculation? Is the data gathered in depth over time or does it represent a brief glimpse of the theory/situation being assessed? Is it safe to generalise from this data? Is the data/theory being described also seen in other sources (triangulation)? Is the data/theory consistent with the experience of the research team and the stakeholder groups (Subject Expert Group and PPIE group)?[27,28]

Formatted: Font: (Default) Aptos, 11 pt, Italic, Complex Script Font: Arial, 11 pt, Italic, English (United Kingdom)

Formatted: Font: (Default) Aptos, 11 pt, Italic, Complex Script Font: Arial, 11 pt, Italic, English (United Kingdom)

Formatted: Font: (Default) Aptos, 11 pt, Italic, Complex Script Font: Arial, 11 pt, Italic, English (United Kingdom)

Formatted: Font: (Default) Aptos, 11 pt, Italic, Complex Script Font: Arial, 11 pt, Italic, English (United Kingdom)

When considering whether to include documents on the basis of rigour and relevance, relevance will be valued over rigour. However, we will ensure that subsequent work endeavours to substantiate the contribution of any data that is not sufficiently rigorous and, when reporting the results of the realist review, we will ensure that we highlight any areas that are supported by data of questionable rigour.

Reviewer: 2
Prof. Scott Weich, University of Sheffield
Comments to the Author:
Thank you for asking me to look at this review protocol.
This is an important study and the protocol will be of interest. However, I think there are elements that could be clarified and strengthened.

In particular, it took me a little while to appreciate that the focus of the study (and the realist question) is patient involvement in risk assessment and management. There's a bit of slippage in terminology and words like 'collaboration' and 'involvement' are used interchangeably. This is an very important topic and I think the early parts of the manuscript might be more impactful if the focus and aims were made a little clearer.

Our response:

This is a helpful observation. We have now ensured that we use the term "collaboration" throughout, rather than involvement. We have also amended the introduction to clarify the focus earlier, which we agree is an improvement.

Introduction and background, page 3, paragraph 3:

To align with the goal of the NHS Long Term Plan to maximise patient-centred care, autonomy and involvement in decision-making, Considering the pivotal role that risk assessment and management play in progression through the secure mental health pathway, it is important that patients are offered the opportunity to engage in meaningful collaboration with professionals in creating those risk assessments and management plans. there is a need for special consideration of how collaboration in risk assessment and management planning can be achieved in this complex pathway. This importance is highlighted by the goal of the NHS Long Term Plan being to maximise patient-centred care, autonomy and involvement in decision-making, [6] *and in the context of the UK's Independent Review of the Mental Health Act* [7] *which set out a number of recommendations for frameworks to become more responsive to patients' needs and wishes, protect patients' rights, and improve patients' ability to contribute towards choices even in restrictive circumstances.*

There is a need for special consideration of how such collaboration in risk assessment and management planning can be achieved in the complex pathway of secure mental health services. Optimising patient-centred care in secure settings, by making assessment of risk and aligned management decisions a collaborative process, has specific challenges given the potential issues with insight, capacity and other factors.[8] *This is complicated further by various other individual and environmental contextual factors that may impact collaboration, as well as the involvement of a combination of agencies which may have competing needs and priorities. various other and environmentalcontextual factors that, as well as the involvement of a combination of agencies.*

Likewise, the PPIE aspects of the research could be a little clearer (and more consistent) especially as this feels like a real strength of the research.

Our response:

We have revised and expanded the PPIE section to make this element clearer and to provide more detail about its formation and activities. We agree with the reviewer that this is one of the key strengths of the work and so have afforded this more space in the revised manuscript.

Methods and analysis, Patient and public involvement, page 6, paragraph 1:

Patient and public involvement

Formatted: Font: (Default) Aptos, 12 pt, Italic, Complex Script Font: Arial, 12 pt, Italic, English (United Kingdom)

Patient and public involvement and engagement (PPIE) is embedded throughout by the formation of a PPIE group consisting of 12 people with lived experience, either current or past, of secure mental health services as patients and carers.

Participants were identified through existing Rethink Mental Illness channels, national lived experience and clinical networks and from other projects in secure services. All participants received an information sheet, gave informed consent and participated in informal calls to assess wellbeing and readiness prior to their joining the group. The PPIE group includes people who have entered the secure pathway via different routes, including transfer from prison, and there is a good geographical spread across England.

The PPIE group will meet online approximately three times for two hours throughout the review process. There will be two facilitators and one note-taker from Rethink Mental Illness. Meetings will begin with a group agreement to remind people of safety and for the comfort of participants and for safeguarding purposes. Optional debrief meetings will be held two to three days after group meetings to allow participants to make any further contributions and to check on wellbeing,

Agreements have been made with the clinical teams of participants in hospital that ward staff can support the patient's participation in PPIE meetings by arranging for secure ward laptops to be used in private areas of the ward. Communication from the co-ordinating team at Rethink Mental Illness therefore goes to both the group member, if they have an email address, as well as to the contact on the ward, who is asked to print out any pre-reading materials, such as agendas, previous minutes, or background reading for the meeting.

The PPIE group will support the research team in refining the evidence search by giving feedback on the list of search terms, reviewing and developing 'If…then…' statements and contributing to and sense-checking Context, Intervention, Mechanism and Outcome (CIMO) configurations. They will offer perspectives on the emerging themes and where to target additional searches, and to support the team in developing and refining the Programme Theory (PT), which aims to explain how and why collaborative risk assessment and management works for different groups of people. They will also be able to contribute to the research after meetings by providing feedback via email. Finally, once the PT is finalised and the review process is complete, the PPIE group will also input into the co-production of outputs and their dissemination.

In terms of describing the study methods, I note that the review appears to conclude with evidence synthesis rather than further iteration of the PTs together with stakeholders and lived experience experts. If that's the case, then this is fine. But I wondered if this is planed but not fully described here?

Our response:

We agree that we can describe our plans more clearly here. We intend to consult the stakeholder groups (the subject expert group and the PPIE group) at each stage of the programme theory development, including when the final draft is formulated. We have expanded the concluding stage of the review to explain this further.

Study Design, Stage 4, page 13, paragraph 3:

As the programme theory develops, we will consult the Subject Expert Group (SEG) and the PPIE group at regular intervals for the purposes of sense-checking the theory as it compares to their experience. As an example of this, ~~F~~for the Subject Expert Group (SEG) and PPIE group meetings, the research team will devise clear CIMO statements, comprising of 'if' – 'then' logic. Effort will be made to make these statements understandable in the context of peoples' experience. This will enable members to deliberate and contribute further suggestions for refinement of the CIMOs and PT.

If needed, we will conduct further searches to fully understand and develop the relationships between the contexts, mechanisms, and outcomes identified. Throughout the analysis we will move iteratively between identifying subsections of data as context, intervention, mechanism, or outcome, developing the CIMOCs, refining the overall programme theory, and conducting further searches. In the last ~~is~~ stage of the review process, we will ensure that we consult the SEG and the PPIE group when we have the final draft of the programme theory. They will have been consulted at each stage of the programme theory development; when forming the initial programme theory, when defining the review search terms and when developing CIMO configurations using if-then statements. However, this final consultation will allow the SEG and PPIE groups to fine tune the programme theory ~~when developing CIMOCs and adding them to the initial PT~~ to ensure that it~~these decisions~~ align with the experiences of both those working in practice and those receiving care.

One further reflection is that it's not altogether clear what's meant by 'secure' services, and which of the NHS and privately provided services might be in scope? Does this include only medium security services, or does it extend to high and/or low security forensic services?

Our response:

We have expanded our definition of secure services, as indeed this covers multiple clinical settings which are worthy of specifying.

Introduction and background, page 2, paragraph 2:

The secure mental health pathway in England is comprised of the secure hospital system, which in England exists across three security levels (low, medium and high) and consumes around a fifth of the annual mental health budget [1] and community forensic mental health teams. This pathway includes independent sector secure hospitals who may be commissioned to provide care as part of NHS-led Provider Collaboratives.[2]

Formatted: Indent: Before: 0.5"

Finally, I wonder if the limitations of the research might extend beyond a single bullet point. Realist reviews have much to recommend them but they are not without their critics.

Our response:

Thank you, we have amended the strengths and limitations of the study to include a fuller consideration of the limitations of the realist review and have reproduced this section in our response to Editors' comments above.

Reviewer: 3
Dr. Jantien Van Berkel, Utrecht University, Wageningen University & Research
Comments to the Author:
I appreciate the opportunity to review this study protocol and would like to commend the authors for developing an interesting and well-structured research plan. The realist approach chosen for this study is particularly promising, as it has the potential to generate relevant and nuanced findings regarding secure mental health services. I have only minor remarks, which I hope will be helpful in further refining and preparing the study.

Introduction

Terminology Consistency:
The introduction would benefit from greater consistency in terminology, particularly concerning the description of secure mental health services. The title refers to "secure mental health services," whereas the introduction mentions "secure psychiatric services." Later, it becomes clear that secure services encompass different types of facilities. Ensuring clarity and consistency in these terms will help the reader better understand the scope of the study.\

Our response:

We thank the reviewer for also highlighting the benefit of clarifying this for the reader, and kindly refer to our response to the penultimate comment from reviewer 2 above. We also now use the term "secure psychiatric services" throughout for consistency.

Strengthening the Justification for a Realist Review:

The last three paragraphs of the introduction argue why a realist review is necessary. While I agree that a realist approach is well-suited to this topic and will yield valuable insights, the reasoning as presented could be made more robust. The authors mention that systematic reviews exist but that evidence remains scarce. However, scarce evidence alone does not necessarily justify conducting another review, as a review does not expand the empirical evidence base per se. Instead, a realist review provides a different analytical lens to examine existing literature, helping to unravel underlying mechanisms, relevant subgroups, and contextual factors. If the literature does not provide these insights, the review will help identify knowledge gaps in these areas. The justification should focus on the need for a nuanced understanding of how interventions work, under what circumstances, and for whom. Additionally, it is worth highlighting that the interventions studied are complex, as are the systems in which they are embedded. The authors might consider referencing Malcolm Williams' work on complex systems and realism to strengthen this argument.

Our response:

We thank the reviewer for this considered reflection. We have chosen to implement this refocusing of justification mainly by revising the final paragraphs of the introduction to better highlight the specific value of using a realist methodology to address this clinical scenario and research question. As part of this we now include the following reference:

Commented [DW1]: Presume on of the refs is now a Malcolm Williams one - put the citation here (and need to add if you haven't).

Introduction and background, page 4, paragraph 4:

> *There have been two systematic reviews of patient collaboration in risk assessment in secure settings, which have identified promise in terms of the feasibility of implementing collaborative risk assessment, and some positive outcomes arising from the inclusion of patients in risk assessment and management.*[12,16] ~~*However, the*~~*However, these reviews focused narrowly on violence risk assessment and quantitative studies and emphasised patient collaboration only (rather than also considering families and carers). Of note, two studies found that around 70% patients residing in secure settings have contact with carers, and around 56% of carers are involved in discharge planning.*[17,18] *The specific importance of carer collaboration throughout secure mental*

health service involvement has previously been highlighted,[19] and a review of evidence pertaining to carer collaboration in risk assessment specifically remains a key evidence gap. Finally, systematic reviews excel in synthesising evidence for or against an intervention and, whilst this is valuable, they are unable to delve more deeply into why or how the intervention might work or how it might work differently for different groups. Given the inherent complexity of patient collaboration in risk assessment and management, a realist review is well suited to this question.[20] Rather than assessing overall efficacy of an intervention, realist reviews focus instead on understanding the underlying mechanisms and contexts through which an intervention might work and on differentiating how these mechanisms and contexts might operate within different subgroups of patients.[21] By adopting this approach, in conjunction with the wide stakeholder involvement embedded in the work, it can achieve outputs that are both theoretically anchored and practically relevant.-

Methods and Analysis
Subheading "Patient and Public Involvement":
I found this heading somewhat surprising, as the study does not only involve patients and the public but also experts, including policymakers and researchers. A more inclusive term, such as "Stakeholder Involvement" or "Stakeholder Consultation," may better reflect the breadth of participation. Furthermore, if not already included, I recommend involving frontline mental health professionals, as their unique perspective could significantly enrich the study.

Our response:

Thank you, we agree this distinction is worth specifying, and we have chosen to do so using a new subheading of “Stakeholder Involvement”, and then separating discussion of this into “Patient and public involvement” and “Subject expert group”. The Subject Expert Group section now includes further information about the formation of this group, including its inclusion of frontline clinicians how it was recruited and its activities.

Methods and analysis, Stakeholder involvement, Subject Expert Group, page 8 paragraph 1:

Subject Expert Group (SEG)

The Subject Expert Group is comprised of 24 stakeholder representatives. It includes representation of all levels of hospital security, community forensic services, women's services, specialist settings including for intellectual disability and autism, NHS and independent sector providers, The Royal College of Psychiatrists, British Psychological Society, Ministry of Justice and First Tier Tribunal. The group includes a range of multiprofessional frontline clinicians, in addition to our PPIE lead and an additional carer

Formatted: Font: (Default) Aptos, Italic, Complex Script Font: Italic, English (United Kingdom)

Formatted: Font: (Default) Aptos, 11 pt, Complex Script Font: 11 pt, English (United Kingdom)

Formatted: Font: (Default) Aptos, 11 pt, Italic, Complex Script Font: 11 pt, Italic, English (United Kingdom)

Formatted: Font: (Default) Aptos, 11 pt, Italic, Complex Script Font: Arial, 11 pt, Italic, English (United Kingdom)

Formatted: Font: (Default) Aptos, 11 pt, Italic, Complex Script Font: Arial, 11 pt, Italic, English (United Kingdom)

Formatted: Font: (Default) Aptos, 11 pt, Italic, Complex Script Font: Arial, 11 pt, Italic, English (United Kingdom)

Formatted: Font: (Default) Aptos, 11 pt, Italic, Complex Script Font: Arial, 11 pt, Italic, English (United Kingdom)

Formatted: Font: (Default) Aptos, 11 pt, Italic, Complex Script Font: Arial, 11 pt, Italic, English (United Kingdom)

representative. The group was initially formed via the professional network of the research team and was subsequently iteratively populated according to gaps identified by the group itself in initial meetings.

The group will meet together with the research team approximately three times throughout the realist review process, with the possibility of ad hoc meetings/workshops taking place as required. The SEG will provide input during key stages of the review process including but not limited to: (i) the initial formation of the programme theory, to assess its validity when compared to current clinical practice, (ii) development of literature search terms and suggestions for additional searches as needed, (iii) processes of data synthesis including the development of "if…then" statements and development of context-intervention-mechanism-outcome configurations, (iv) development of the final programme theory and fine tuning the theory to accurately reflect current practice, (v) plans for dissemination of the results of the review.

Formatted: Font: (Default) Aptos, 11 pt, Not Bold, Italic, Complex Script Font: 11 pt, Not Bold, Italic, English (United Kingdom)

Ethical Considerations:
I did not find explicit ethical considerations regarding the involvement of patients, the public, and experts. Given the participatory nature of the study, it would be beneficial to outline the ethical framework governing these interactions, including issues such as informed consent and data protection.

Our response:

This is helpful to highlight, which we have done in the substantially expanded PPIE section included above in the response to Reviewer 2. This now provides much fuller details of the safeguards, governance and practical conduct of this activity.

Details on Study Phases:
Under "Study Design," in the Methods and Analysis section, more concrete details are needed for Phases 2, 3, and 4. It is currently unclear what specific steps will be taken, and how relevance and rigour will be assessed for example.

Our response

We have made significant amendments to the methods section (Phases 2,3 and 4) in line with these comments along with those provided on the pdf provided. These include 1) removal of references to the iterative searches from Phase 2 (these happen after data extraction and so are best discussed there), 2) an expansion of the sections in Phase 3 about rigour and relevance (the methodology now explicitly states how we will assess each of these elements), 3) an expansion of the final stages of data extraction and synthesis in phase 4 (including how we will involve the PPIE and SEG in this final validation of the programme theory and including the

specific changes mentioned in the pdf around how we handle information that does not fit in with existing CIMOCs). We include below from convenience the most substantially revised section, including relevance and rigour:

Stage 3, page 11, paragraph 3:

> *During the full text screening, the lead reviewer will assess each document according to the principles of relevance and rigour~~rigour and relevance~~.*[23] *Relevance in realist methodologies is defined as "whether [the data] can contribute to theory building and/or testing".* [21] *In making decisions about whether a particular document is relevant, the reviewer will ask two questions.* [27] *Firstly they will ask whether the document is relevant to the topic of collaborative risk assessment and management in secure mental health services. Secondly they will ask whether the document provides evidence that is relevant to the development of the programme theory. The first will be determined during full text screening, whereas the second question will be reassessed as the programme theory develops. This reassessment is necessary as previously excluded evidence may become relevant during theory development. Any papers that might possibly be relevant in future will be assigned to the "reserve list" to allow for this reassessment if needed. The RAMESES quality standards for realist reviews define rigour as '"whether the method used to generate that particular piece of data is credible and trustworthy".*[21] *The RAMESES guidelines do not recommend any particular checklist, however, when considering text or data for inclusion the following questions will be asked: Is the data likely to be biased? Is the data/theory described in the text critically analysed? Does the data/theory derive from real-world examples or is it based on theoretical speculation? Is the data gathered in depth over time or does it represent a brief glimpse of the theory/situation being assessed? Is it safe to generalise from this data? Is the data/theory being described also seen in other sources (triangulation)? Is the data/theory consistent with the experience of the research team and the stakeholder groups (Subject Expert Group and PPIE group)?* [27,28]
>
> *When considering whether to include documents on the basis of rigour and relevance, relevance will be valued over rigour. However, we will ensure that subsequent work endeavours to substantiate the contribution of any data that is not sufficiently rigorous and, when reporting the results of the realist review, we will ensure that we highlight any areas that are supported by data of questionable rigour.* ~~*Rigour is defined as the extent to which the conclusions drawn by the researchers are supported by the data and relevance is the extent to which the document can be reasonably judged to contribute to the PT.*~~

We note that the reviewer suggests that a flow chart of the process would be helpful – we agree, and would kindly direct the reviewer to the figure included with the paper. We are assuming that this was simply overlooked by the reviewer (given that it was submitted as a separate file as per journal requirements), rather than the reviewer suggesting another, additional flow chart. We have copied the figure in question below for convenience.

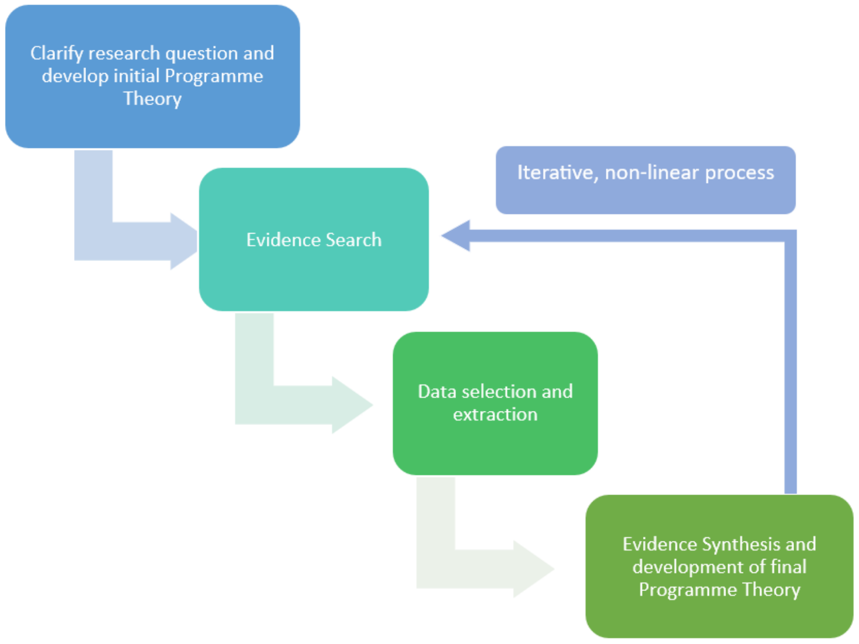

Additionally, given the likely high number of articles retrieved, the title and abstract screening process will be time-consuming. The authors should consider discussing potential strategies for managing this workload, such as using screening tools like Rayyan.

Our response:

We have now clarified that we will be using RAYYAN software for the title and abstract screening phase.

Study design, Stage 3, paragraph 6:

> *To determine whether a document should be included in the review, two members of the research team will screen the titles and abstracts of all documents retrieved by the searches. The title and abstract screening process will be supported through use of RAYYAN software.*

Strengths and Limitations

To further reflect on the study protocol, I suggest discussing the strengths and limitations (currently only outlined in the abstract) in some more detail.

Strengths: One of the major strengths of this study is its use of a realist approach, which allows for a deeper exploration of the mechanisms driving outcomes in secure mental health services. This approach is particularly useful given the complexity of the interventions and the systems in which they operate. Additionally, the inclusion of multiple stakeholder perspectives—including patients, professionals, and policymakers—enhances the study's potential to generate findings that are both theoretically and practically relevant.

Our response:

This is a helpful suggestion which mirrors the thoughts of reviewer 2 regarding better highlighting the specific methodological strengths of the realist review for this topic. We have addressed this in our revised introduction, reproduced fully in the response to reviewer. We have included the most relevant excerpt again below.

Introduction and background, page 4, paragraph 4:

> *Rather than assessing overall efficacy of an intervention, realist reviews focus instead on understanding the underlying mechanisms and contexts through which an intervention might work and on differentiating how these mechanisms and contexts might operate within different subgroups of patients.*[20] *By adopting this approach, in conjunction with the wide stakeholder involvement embedded in the work, it can achieve outputs that are both theoretically anchored and practically relevant.-*

Limitations: One possible limitation is the reliance on existing literature, which may not always provide sufficient details on mechanisms and contextual factors. While the authors acknowledge this by incorporating stakeholder consultation, further discussion on how potential gaps in the literature will be addressed could be beneficial. Additionally, given the large number of studies likely to be screened, the feasibility of the review in terms of time and resources should be considered, along with potential mitigation strategies.

Our response:

Regarding gaps, in the section Stage 4: Evidence synthesis and development of final programme theory we now specify that where gaps in evidence exist in secure settings for specific parts of the theory, we will incorporate transferrable evidence from relevant related clinical settings.

> *Where there are any gaps in the evidence from secure mental health settings we will utilise the "reserve list", which will hold papers that provide relevant insights into collaborative risk assessment in general adult psychiatric services. When reporting the programme theory, any areas which rely on papers from the reserve list will be highlighted.*

Further, we have added a clearer explanation that the review will feed into a mixed methods study (survey and interviews) which will target gaps in understanding.

Ethics and dissemination, page 14, paragraph 2:

> *The development of the PT will also highlight any gaps in the literature, which can be addressed in subsequent mixed methods work to be undertaken by the research team with surveys and interviews We will also aim to publish the review in a peer-reviewed journal article and present findings at relevant professional conferences.*
>
> *The next stages of the work will use mixed methods to further develop the findings of the review. This mixed methods work package will be developed in conjunction with the PPIE group and the Subject Expert Group and will , to assess the current usage of collaborative risk assessment and management strategies in England and the observed contexts, mechanisms and outcomes associated with these practices.*

We have added a reference to the consideration of feasibility and risk mitigation, which was examined and justified as part of the competitive grant application process for the work. This is included at the end of the introduction where the project start and end dates are listed.

I have provided further specific comments within the uploaded PDF. I appreciate the efforts of the authors in developing this study protocol and look forward to seeing its results.

Our response:

We are grateful to the reviewer for the additional comments on the pdf of the manuscript, which have all been addressed.
